# 560 Utilization of Alternate Hinge for Static Progressive Shoulder Orthosis: A Case Study

**DOI:** 10.1093/jbcr/irad045.156

**Published:** 2023-05-15

**Authors:** Andria Martinez, Renee Warthman, Kevin Foster, Derek Murray, Karen Richey

**Affiliations:** Arizona Burn Center at Valleywise Health, Peoria, Arizona; Arizona Burn Center at Valleywise Health, Phoenix, Arizona; AZ Burn Center at Valleywise Health, Phoenix, Arizona; Arizona Burn Center Valleywise Health, Phoenix, Arizona; Arizona Burn Center Valleywise Health, Phoenix, Arizona; Arizona Burn Center at Valleywise Health, Peoria, Arizona; Arizona Burn Center at Valleywise Health, Phoenix, Arizona; AZ Burn Center at Valleywise Health, Phoenix, Arizona; Arizona Burn Center Valleywise Health, Phoenix, Arizona; Arizona Burn Center Valleywise Health, Phoenix, Arizona; Arizona Burn Center at Valleywise Health, Peoria, Arizona; Arizona Burn Center at Valleywise Health, Phoenix, Arizona; AZ Burn Center at Valleywise Health, Phoenix, Arizona; Arizona Burn Center Valleywise Health, Phoenix, Arizona; Arizona Burn Center Valleywise Health, Phoenix, Arizona; Arizona Burn Center at Valleywise Health, Peoria, Arizona; Arizona Burn Center at Valleywise Health, Phoenix, Arizona; AZ Burn Center at Valleywise Health, Phoenix, Arizona; Arizona Burn Center Valleywise Health, Phoenix, Arizona; Arizona Burn Center Valleywise Health, Phoenix, Arizona; Arizona Burn Center at Valleywise Health, Peoria, Arizona; Arizona Burn Center at Valleywise Health, Phoenix, Arizona; AZ Burn Center at Valleywise Health, Phoenix, Arizona; Arizona Burn Center Valleywise Health, Phoenix, Arizona; Arizona Burn Center Valleywise Health, Phoenix, Arizona

## Abstract

**Introduction:**

Management of shoulder burn injuries creates unique challenges as the shoulder girdle is comprised of four joints and has the largest degree of motion in the body. The complexity of arthrokinematics and cutaneokinematics requires creative positioning devices to achieve the desired effect. Shoulder positioning devices for pediatric patients are limited, and devices that allow positioning at end range can be bulky, heavy, and costly. Available hinges for upper extremity devices offer limited utility and do not withstand the tensile forces of burn scar contracture in conjuncture with and prolonged use. This is a report of a single patient for which a locking knee hinge was used to fabricate a custom static progressive shoulder flexion device.

**Methods:**

This case study analyzes the impact of bilateral custom static shoulder flexion orthoses and static progressive orthoses on range of motion in a pediatric patient. Static shoulder flexion orthotics were implemented, and then transitioned to static progressive orthotics. Goniometric measurements were obtained through out hospitalization. Average variance in of range of motion at the glenohumeral joint (GH), and standard deviation of goniometric measurements comparing static and static progressive orthoses were collected.

**Results:**

There was a total of 33 bilateral sets of goniometric measurements. Sixteen sets were collected utilizing static shoulder flexion orthoses. Mean flexion was 149.6⁰ and 154.6⁰ abduction of the left shoulder. Right shoulder flexion and abduction means were 149.3⁰ and 152.8⁰ degrees. There was a standard deviation (STD) of 22⁰ for the left shoulder mean goniometric measurements and STD of 23.2⁰ for the right shoulder. Seventeen sets were collected utilizing static progressive shoulder flexion orthoses. Results revealed a mean of 166.1⁰ and 166.7⁰ for flexion and abduction of the left shoulder. Right shoulder flexion and abduction averages were 169.3⁰ and 170.6⁰ respectively. There was an STD of 8.9⁰ for mean goniometric measurements of the left shoulder and STD of 4⁰ of the right shoulder. Introduction of a locking hinged shoulder flexion orthosis resulted in an increase of 80⁰ left and 90⁰ right shoulder flexion in 24 hours. The patient was able to achieve and maintain an average of 165⁰ of shoulder flexion throughout hospitalization (47 days).

**Conclusions:**

Use of a locking hinged shoulder orthosis allowed for progressive increase in shoulder flexion without neurological symptoms and decreased pain and anxiety related to therapy and orthotic application. Compliance with wearing schedule was increased due to ease of application.

**Applicability of Research to Practice:**

Utilization of locking hinges to create custom static progressive shoulder flexion orthoses may allow therapists to increase and maintain shoulder range of motion in pediatric patients following burn injury.

